# Abundance of bacterial Type VI secretion system components measured by targeted proteomics

**DOI:** 10.1038/s41467-019-10466-9

**Published:** 2019-06-13

**Authors:** Lin Lin, Emmanuelle Lezan, Alexander Schmidt, Marek Basler

**Affiliations:** 1 Biozentrum, University of Basel, CH 4056 Basel, Switzerland; 2 Proteomics Core Facility, Biozentrum, University of Basel, CH 4056 Basel, Switzerland; 3 Present Address: Roche Innovation Center Basel, CH 4070 Basel, Switzerland

**Keywords:** Membrane proteins, Mass spectrometry, Bacterial secretion, Proteomics

## Abstract

The Type VI secretion system (T6SS) is important for bacterial competition as well as virulence in many Gram-negative bacteria and its dynamics and regulation varies significantly between species. To gain insights into the mechanisms regulating T6SS assembly, we apply targeted proteomics to determine the abundance of the key T6SS components in *Vibrio cholerae*, *Pseudomonas aeruginosa* and *Acinetobacter baylyi*. We show that while there are species specific exceptions, the abundance of most components is similar in all three bacteria and ranges from less than hundred to tens of thousands of copies per cell. The comparison of T6SS dynamics and protein abundance in *V*. *cholerae* grown under various conditions suggests that the critical component TssE and the secreted protein VasX are unstable and this diminishes T6SS assembly when protein synthesis is limited. Our quantitative analysis opens possibilities to build realistic models of T6SS assembly and to identify principles of T6SS regulation in various species.

## Abstract (web summary)

Type VI secretion systems (T6SS) are important for bacterial interaction, competition and virulence, but the abundance and assembly of their components is still not well understood. Here, the authors apply targeted proteomics to measure the abundance of T6SS components across different species and conditions.

## Introduction

The Type VI secretion system (T6SS) is present in ~25% of sequenced Gram-negative bacteria, including many pathogens where T6SS is often important for virulence^1–5^, however, T6SS is also involved in bacterial interaction and competition^6–8^. T6SS is composed of a cell envelope-associated membrane complex, which spans both membranes and assembles from TssJ, TssL, and TssM^9–12^. TssK connects the membrane complex to the baseplate that consists of at least TssE, TssF, and TssG^13–15^. VgrG/PAAR spike is in the center of the baseplate and serves as a platform for initiation of the Hcp tube polymerization^2,14,15^; while the TssB/TssC (also called VipA/VipB) sheath assembles around the inner Hcp tube^16–19^. TssA is involved in baseplate assembly and sheath–tube polymerization^20–22^, while in some organisms, membrane-bound TagA prevents excessive sheath polymerization which may destabilize the sheath^23^. Sheath assembles by addition of soluble subunits at the end distal from the baseplate and its length is correlated with the width of the cell^24^. Upon unknown signal, the sheath undergoes rapid contraction, which propels the rigid inner tube with the effectors associated with Hcp or VgrG/PAAR spike into the target cells^17,25^. The disassembly of the contracted sheath is driven by AAA+ ATPases such as ClpV or ClpB^4,17,26–29^.

Dynamics of T6SS assembly varies among species despite conservation of key components. This is most likely due to different transcriptional or post-translational regulation among bacteria, as well as presence and absence of accessory proteins. For instance, in *Pseudomonas aeruginosa*, the activity of H1-T6SS^30^ relies mostly on the posttranslational regulation. Phosphorylation of Fha by the serine–threonine kinase PpkA is required for T6SS assembly, while the phosphatase PppA is responsible for the Fha dephosphorylation^31^. A periplasmic protein TagR and inner membrane complex TagS/TagT are necessary for the activation of PpkA^32,33^. An outer membrane protein, TagQ, likely tethers TagR to the outer membrane^2,32,33^. In addition, H1-T6SS is negatively regulated by TagF independently of Fha phosphorylation^34^. What activates this signaling cascade is poorly understood, however, this pathway is required for subcellular localization of T6SS assembly in response to T6SS activity in the neighboring cell^8^. Furthermore, H1-T6SS can be activated when cells are sensing membrane damage caused by conjugation or membrane-targeting antibiotics, such as polymyxin B^35^, or chelation of outer membrane-bound cations mediated by extracellular DNA or EDTA^36^.

It remains poorly understood how the initiation of T6SS assembly is regulated among different species. In particular, the abundance of most T6SS components in cells remains largely unknown. This question may be addressable with targeted proteomics approaches, such as selected reaction monitoring mass spectrometry (SRM-MS). SRM is a highly sensitive and selective quantitative approach to determine abundances of selected target proteins with high precision and reproducibility^37,38^. It offers a wide dynamic quantification range starting from tens of copies to 10^6^ copies per cell in yeast^38^ or over four orders of magnitude in *Mycobacterium tuberculosis*^39^. Moreover, it allows consistent monitoring of a set of proteins over multiple samples and to determine absolute protein levels directly in samples when combined with spiked-in heavy reference peptides^40,41^.

Here, we apply targeted proteomics using stable isotope dilution (SID) in combination with SRM-MS to quantify the protein abundance of the known T6SS components in three organisms, the H1-T6SS from *P*. *aeruginosa* PAO1, *Vibrio cholerae* 2740-80, and *Acinetobacter baylyi* ADP1. Moreover, we determine the abundance of the *V*. *cholerae* T6SS components under different growth conditions and compare it with the dynamics of T6SS assembly in live cells. The abundance of most components reflects their function in all three bacteria, with as low as a few hundred copies per cell for spike proteins and tens of thousands of copies for sheath components. The comparison of T6SS protein abundance in *V*. *cholerae* grown under various conditions suggests that the baseplate component TssE and the secreted protein VasX are unstable, which greatly reduces the T6SS assembly when protein synthesis is limited.

## Results

### Quantification of abundance of T6SS components

**Fig. 1:**
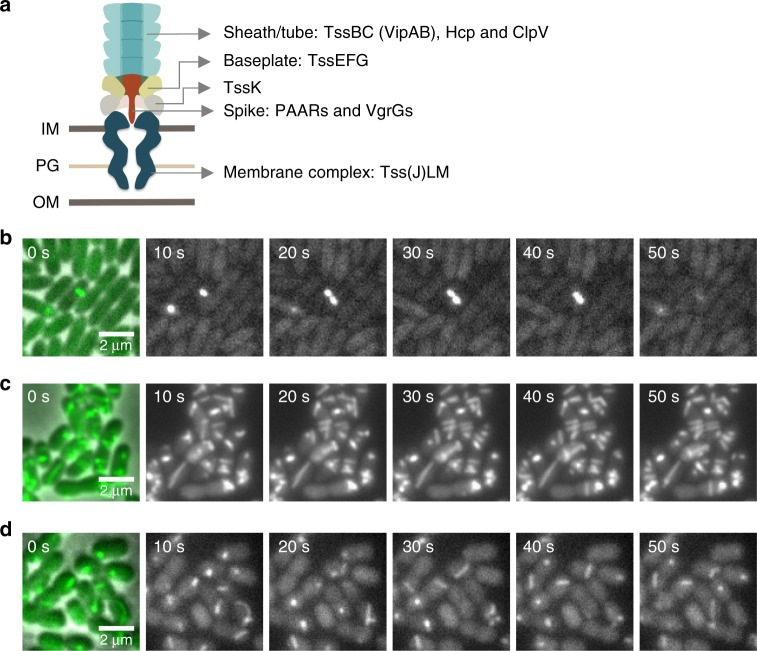
The various T6SS dynamics in different bacteria. **a** The overall structure of T6SS adapted from Nazarov et. al.^14^. **b** Time-lapse imaging of H1-T6SS dynamics in cells of *P*. *aeruginosa* PAO1 (*ΔretS* background). The most left panel shows a merged image of phase contrast and GFP channel (ClpV-GFP); the other panels show the time-lapse series of GFP channel (ClpV-GFP) in an interval of 10 s. **c** Time-lapse imaging of T6SS dynamics in cells of *V*. *cholerae* 2740-80. The most left panel shows a merged image of phase contrast and GFP channel (TssB-msfGFP); the other panels show the time-lapse series of GFP channel (TssB-msfGFP) in an interval of 10 s. **d** Time-lapse imaging of T6SS dynamics in cells of *A*. *baylyi* ADP1. The most left panel shows a merged image of phase contrast and GFP channel (TssB-sfGFP); the other panels show the time-lapse series of GFP channel (TssB-sfGFP) in an interval of 10 s. Scale bar: 2 µm

Most T6SSs are assembled from 10 to 15 conserved structural components including membrane complex and baseplate, as well as sheath/tube (Fig. 1a). Despite the conservation of key components of T6SS, this nanomachine exhibits strikingly diverse dynamics among species. The H1-T6SS from *P*. *aeruginosa* is activated in response to membrane perturbation (Fig. 1b); while cells of *V*. *cholerae* constitutively assemble 3–5 structures that are dynamically positioned within cells without any apparent localization pattern (Fig. 1c). Similarly, *A*. *baylyi* ADP1 cells assemble 1–2 highly dynamic structures (Fig. 1d).

To get insights into the potential mechanisms of regulation of T6SS assembly initiation, we first determined the absolute copy number of proteins in various bacteria grown under conditions that allow for different levels of T6SS activity. We grew *P*. *aeruginosa* PAO1 *ΔretS*, *V*. *cholerae* 2740-80, and *A*. *baylyi* ADP1 to different OD_600_ from 0.5 to 4.0. We counted the bacteria in these cultures using flow cytometry (Supplementary Table 1). The whole cell lysates were then prepared from known number of cells and protein concentrations were measured using BCA assay (Thermo Fisher). This allowed us to estimate that total protein amount is ~280 fg/cell for *P*. *aeruginosa* with an OD_600_ 1.2–1.5, 156–260 fg/cell for *V*. *cholerae* with an OD_600_ 0.5–4.0, and 210 fg/cell *A*. *baylyi* with an OD_600_ 0.8–1.2 (Supplementary Table 1). This is consistent with estimated protein concentrations in the range of 200–300 mg/ml^42^ and volume of a typical bacterial cell of ~1 µm^3^. These measured values were then used to estimate the number of cells across all cell lysates grown to similar OD_600_ by measuring concentration of total proteins in each sample. The errors of such estimations come from both cell number count by FACS and protein content determination by BCA and are 5.02%, 7.45%, and 7.34% among triplicates for *P*. *aeruginosa*, *V*. *cholerae*, and *A*. *baylyi*, respectively. To minimize additional errors introduced during sample preparation, we used a recently established protocol that demonstrated highly efficient and reproducible protein extraction and digestion from bacterial cells, in particular for membrane proteins^43^.

**Fig. 2:**
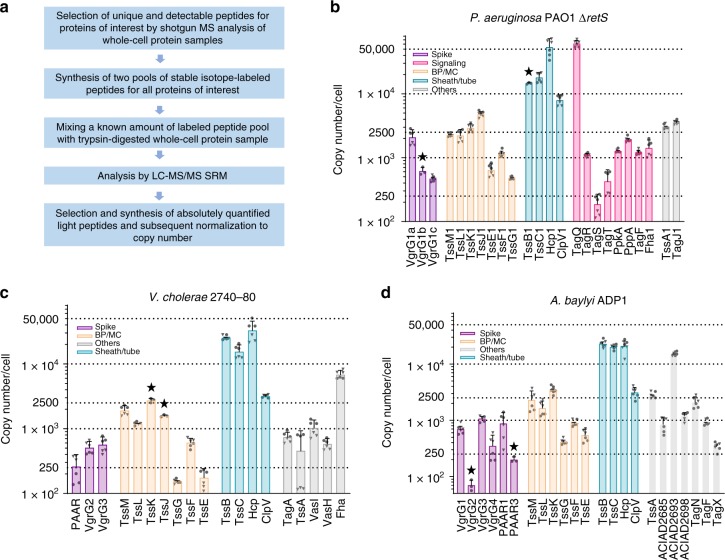
Abundance of T6SS components in different bacteria. **a** The overall experimental procedure for determining the abundance of proteins in this work. **b** The absolute copy number of each selected component of H1-T6SS in whole cell lysate of *P*. *aeruginosa* PAO1 (*ΔretS* background) grown to OD_600_ 1.2–1.5. **c** The absolute copy number of each selected component of T6SS in whole cell lysate of wild-type *V*. *cholerae* 2740–80 grown to OD_600_ 1.2–1.5. **d** The absolute copy number of each selected component of T6SS in whole cell lysate of wild-type *A*. *baylyi* ADP1 grown to OD_600_ 1.2–1.5. **b**–**d** The quantification of heavy peptides from the peptide mixes were determined using corresponding light quantified peptides in triplicates. All abundance measurements were performed in three independent biological experiments. Bar graph shows the average of copy number determined by two peptides (individual measurements shown with dots for peptide 1 or triangles for peptide 2), with error bar as the SD between measurements from both peptides if available; otherwise, SD between biological replicates is shown (highlighted with a star). Note the *y*‐axis is in Log_10_ scale. All measurements are available in Supplementary Data 1. Source data are provided as a Source Data file

We then applied SID-SRM-MS using the combination of stable-isotope-labeled peptide pools and precisely quantified light peptides (JPT peptides) to quantify all the structural proteins, as well as regulatory components in all three bacteria (Fig. 2a, Supplementary Data 1, see the section “Methods” for details). For 60 out of 66 proteins, copy numbers were determined from two independent peptide measurements (Supplementary Data 1). For determination of peptide concentrations in the heavy peptide pool, 123 out of 126 selected peptides displayed <30% coefficient variation during MS analysis (Supplementary Data 1). Moreover, in this set of assays, the measurement of 121 out of 126 peptides displayed <20% variation across three biological replicates (Supplementary Data 1), indicating that the measurements were highly reproducible.

Overall, our results show that abundance of T6SS components range from 10 to 10^5^ copies per cell (Fig. 2b–d). The abundance of TssB (VipA), TssC (VipB), and ClpV in *V*. *cholerae* (Fig. 2c) were approximately 25,000, 15,500, and 3100, respectively, which is in a reasonable agreement with the previously reported copy number 27,000, 26,000, and 3500, respectively, estimated by semi-quantitative immunoblotting^26^. In addition, comparison of the abundance of T6SS components in *P*. *aeruginosa* expressing or lacking RetS shows that most proteins except TagS and VgrG1c are about 2–6-fold more abundant in RetS-negative strain (Supplementary Fig. 1; Supplementary Data 6), which is in agreement with previously observed *retS*-dependent regulation of transcription^30,44^.

### Abundance mostly correlates with the stoichiometry of T6SS

The current model of T6SS assembly predicts certain stoichiometry for T6SS components. A single T6SS structure is composed of a single PAAR protein, three VgrG proteins, 5–12 copies of components forming membrane and baseplate complex, however up to thousand copies of Hcp and TssB/TssC^10,14,16,25,45^. Indeed, for all three analyzed bacteria, sheath proteins (TssB and TssC) were highly abundant (in the range of 10^4^ copies per cell). By contrast, spike proteins, such as PAAR or VgrGs were among the least abundant components, suggesting that availability of these proteins could be limiting the number of T6SS assemblies per cell. Baseplate components were in the range of 10^2^–10^3^ copies/cell; while membrane components were in the range of 1–2 × 10^3^ copies/cell. Interestingly, in all three organisms, the components of the membrane complex were more abundant than components of the baseplate (Supplementary Data 1; Fig. 2b–d).

Overall, the relative protein abundance was conserved among all three bacteria, however, some significant differences were observed. For instance, in *P*. *aeruginosa*, Fha1 had low abundance, while the homolog in *V*. *cholerae* is almost four times more abundant (1500 copies and 7000 copies per cell in *P*. *aeruginosa* and *V*. *cholerae*, respectively). While Fha has been shown to be essential for the assembly of T6SS in *V*. *cholerae*, its function remains unclear^46^.

We included the species-specific accessory components in our analysis to get insights into their possible roles in T6SS assembly. The post-translational modification of Fha1 by TagQRST-PpkA pathway is required for assembly of H1-T6SS in *P*. *aeruginosa*. TagR/TagS/TagT as well as PpkA/PppA/Fha were detected in the range of 10^2^–10^3^ copies per cell (Supplementary Data 1). Strikingly, TagQ, which is the outer membrane protein, was the most abundant component (~61,000 copies per cell), while TagR, which binds TagQ, is present only in ~1000 copies per cell. TagS (~200 copies) and TagT (~400 copies) are present at very low copy number, at least seven times less than components of the membrane complex.

Several components of *A*. *baylyi* ADP1 T6SS have currently unknown function, in particular ACIAD2685, ACIAD2693, and ACIAD2698. While ACIAD2693 and ACIAD2698 were found to be dispensable for T6SS function, ACIAD2685 is required for efficient T6SS assembly initiation^47^. Interestingly, ACIAD2685 is present in only about 800 copies per cell. This is similar to another protein TagX (~350 copies), which is predicted to be an enzyme that cleaves peptidoglycan^48^ likely to initiate membrane complex assembly similarly to what was shown for MltE, a transglycosylase in enteroaggregative *E*. *coli*^49^. Together with very low copy number of certain VgrGs and PAAR protein, ACIAD2685 and TagX could be involved in regulation of the frequency of T6SS assembly.

### Change of abundance in response to growth phase

**Fig. 3:**
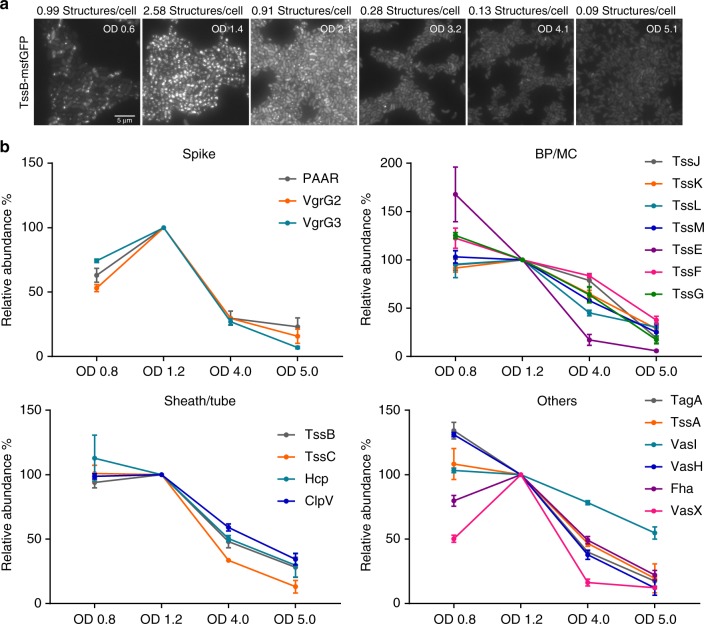
Change in abundance of *V*. *cholerae* T6SS proteins during growth phases. **a** The sheath assembly was monitored by TssB-msfGFP localization during different growth phases. Representative images of GFP channel are shown. Time-lapse movies with a larger field of view can be found in Supplementary Movie 1. The average number of sheaths is indicated above the images. Scale bar: 5 µm. **b** The relative abundance levels of each component during four growth phases (OD_600_ ~0.7, ~1.2, ~4, and ~5, respectively). Note the *y*‐axis is shown as % abundance related to the protein levels at T2 (OD_600_~1.2). The error bar represents the standard deviation of measurements (*n* = 3 biologically independent samples). Plots were grouped into four subsets: spike proteins, baseplate/membrane complex, sheath/tube proteins, and other components. Individual plots are shown in Supplementary Fig. 2. The detailed quantification for this figure can be found in Supplementary Data 2. Source data are provided as a Source Data file

We tested if changes in T6SS activity correlate with changes in protein abundance under varying growth conditions. The observed number of T6SS assemblies per cell increases in *V*. *cholerae* with OD_600_ from ~0.6 with ~1 structure per cell on average to early stationary phase (OD_600_ 1.2–1.5) with 2.58 structures per cell on average. It is worth noting that when the T6SS assembly is high in the cell (above three per cell), measured number of sheath assemblies is likely an underrepresentation of the actual number of sheath assemblies in the cell, as many sheaths would not be resolved using conventional light microscopy. Therefore, it is likely that more than three structures assembled on average at OD_600_ 1.2–1.5. Such high T6SS activity is maintained till OD_600_~2. When cells grow to OD_600_ above 2, the number of T6SS assemblies decreases to about 1 sheath structure per cell. Moreover, at OD_600_ above 4, cells become significantly smaller and the T6SS is mostly inactive (Fig. 3a; Supplementary Movie 1).

We applied the SID-SRM-MS to samples collected from four representative growth stages (OD_600_~0.8, OD_600_~1.2, OD_600_~4, and OD_600_~5). By calculating the copy number per cell as explained above and normalizing to the most active stage of T6SS (Supplementary Data 2, OD_600_~1.2), we compared the protein abundances for all measured T6SS components (Supplementary Data 2; Fig. 3b). When cells transitioned from early exponential phase (OD_600_~0.7) to late exponential or early stationary phase (OD_600_~1.2), concentrations of membrane complex components, as well as TssB and TssC remained unchanged or slightly increased. Notably, during this transition, all baseplate components as well as Hcp showed slight decrease in abundance (Fig. 3b). Interestingly, spike proteins (PAAR and VgrGs), especially VgrG2 showed a striking increase in abundance (Supplementary Fig. 2; ~50%; *p* ≤ 0.01, Tukey’s multiple comparisons test used for all comparisons) when cells grew from early to late exponential phase; while protein levels of PAAR increased by 35% (Fig. 3b; Supplementary Fig. 2; *p* ≤ 0.01). This correlated with the increase of the number of sheath assemblies observed by live-cell imaging (Fig. 3a; Supplementary Movie 1). Levels of most proteins remained unchanged during this transition; Fha exhibited a slight increase, while VasH and TagA showed a slight decrease (Fig. 3b; *p* ≤ 0.05). Interestingly, the level of an effector protein VasX also increased significantly during this phase (Supplementary Fig. 2; 50%; *p* ≤ 0.001).

After the OD_600_ increased to ~4, all components showed significant decrease in their abundance. In particular, TssE and VasX decreased by 85% (Supplementary Fig. 2; *p* ≤ 0.01 and *p* ≤ 0.0001, respectively); while VgrG2, VgrG3, and PAAR decreased by ~70% (Supplementary Fig. 2; *p* ≤ 0.05). Overall, the change observed in the protein levels was in good agreement with live-cell imaging (Fig. 3a; Supplementary Movie 1). In addition to the core components, we also analyzed the change in relative abundance for effector and immunity proteins (Supplementary Fig. 4A) and, with the exception of TsiV3, we observed a similar trend of change as for the structural or regulatory components of T6SS.

### Change of abundance upon protein synthesis inhibition

**Fig. 4:**
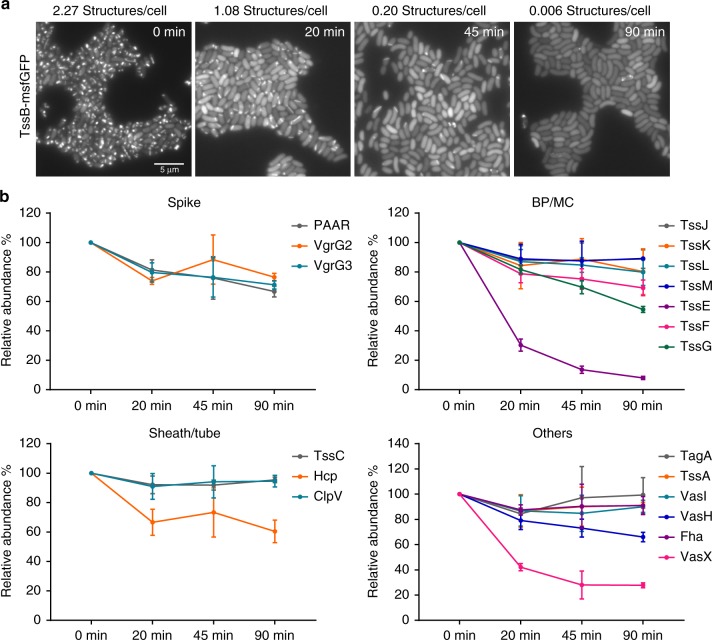
Change in abundance of *V*. *cholerae* T6SS proteins upon chloramphenicol treatment. **a** The sheath assembly was monitored by TssB-msfGFP localization after 1 mg/ml of CAM treatment. Representative images of GFP channel are shown. Time-lapse movies with a larger field of view can be found in Supplementary Movie 2. The average number of sheaths is indicated above the images. Scale bar: 5 µm. **b** The relative abundance levels of each component at four time points following the CAM treatment (0, 20, 45, and 90 min, respectively). Note the *y*‐axis is shown as % abundance normalized to TssB related to the protein levels at T0 (0 min; pre-treatment). The error bar represents the standard deviation of measurements (*n* = 3 biologically independent samples). Plots were grouped into four subsets: spike proteins, baseplate/membrane complex, sheath/tube proteins, and other components. Individual plots are shown in Supplementary Fig. 3. The detailed quantification for this figure can be found in Supplementary Data 3. Source data are provided as a Source Data file

To determine mechanisms responsible for decrease of T6SS activity when expression is downregulated, we analyzed the abundance of T6SS components of *V*. *cholerae* exposed to a high dose of chloramphenicol (1 mg/ml). We examined the morphology of cells after up to 90 min treatment and observed that most cells remained intact (Supplementary Fig. 7). Within 20 min of the protein synthesis inhibition, we observed a dramatic decrease in T6SS activities (from more than 2.3 structures per cell before treatment to only 1 structure per cell). Forty-five minutes after the treatment, only 1 structure can be observed in 5 cells on average. Ninety minutes after the treatment, T6SS assembly was almost completely abolished (Fig. 4a; Supplementary Movie 2). Since we observed no significant decrease of TssB-sfGFP signal by live-cell imaging, we first normalized the abundance of all components to TssB and then compared these to the levels measured before chloramphenicol treatment (Supplementary Data 3; Fig. 4b).

Interestingly, within only 20 min after chloramphenicol treatment, we observed 70% decrease in the abundance of TssE (Supplementary Fig. 3; *p* < 0.0001, one‐way ANOVA with multiple comparison using Tukey correction), as well as smaller decrease of abundance of other baseplate components (Fig. 4b; Supplementary Fig. 3). Ninety minutes after the treatment, TssE abundance dropped below 10%. By contrast, abundance of membrane components remained largely unchanged throughout the treatment (Fig. 4b). The secreted components such as Hcp, VgrG3, and VgrG2, as well as PAAR exhibited a gradual decrease during the treatment (Fig. 4b). TssC, TagA, TssA, VasI, ClpV, and Fha decreased <15% after 90-min treatment, but VasH decreased by more than 30% (Fig. 4b). We also compared the relative abundance of effector and immunity proteins following the protein synthesis inhibition (Supplementary Fig. 4B). Like most T6SS components, these proteins remain relatively stable after the treatment with an exception of TsiV1 and Tap1. Interestingly, abundance of the effector VasX decreased by 60% within 20 min of the treatment (Supplementary Fig. 3; *p* < 0.0001). Overall, VasX and TssE showed the fastest and highest decrease in protein abundance after protein synthesis inhibition. This is similar to what was observed during the transition from exponential to stationary phase (Fig. 3b).

To test the stability of TssE and VasX in the absence of other *V*. *cholerae* proteins, we overexpressed *tssE* or *vasX* in *E*. *coli* from the pBAD24 vector. We then exposed these cells to the same high dose of chloramphenicol and quantified the abundance of TssE or VasX by SRM-MS analysis. Within 90 min of the treatment, we observed decrease of TssE by ~75% and VasX by 85% (Supplementary Fig. 5; Supplementary Data 7). This suggests that both proteins are generally unstable, however, it is unclear how these proteins are degraded in *V*. *cholerae* or *E*. *coli*.

### TssE and VasX are together essential for the T6SS assembly

**Fig. 5:**
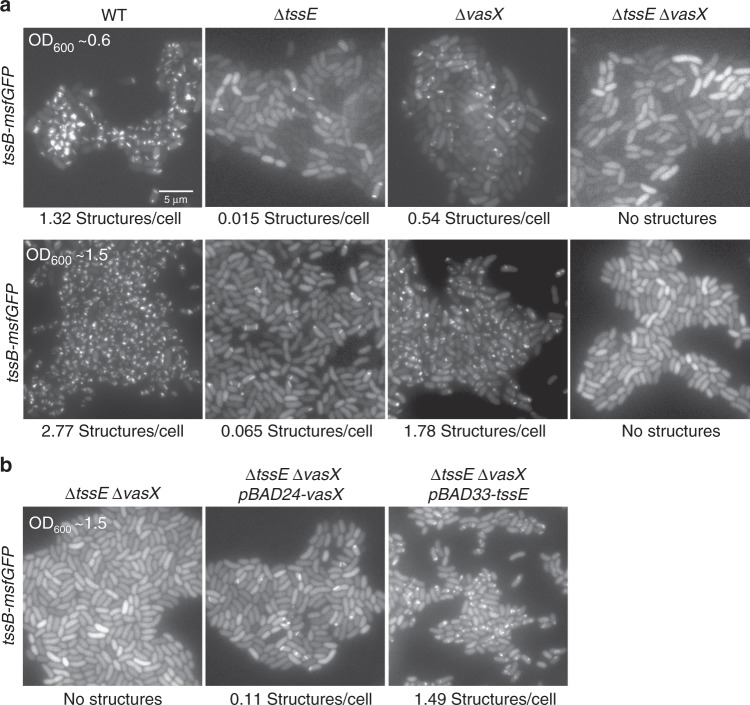
TssE and VasX contribute to the regulation of T6SS assembly. **a** The sheath assembly was monitored by TssB-msfGFP localization in the absence of TssE, VasX, or both. Representative images of GFP channel are shown for each indicated strain (all *tssB-msfGFP* background). The upper panel shows the cells grown to OD_600_ 0.6; the lower panel shows the cells grown to OD_600_ 1.5. The average number of sheaths is indicated below each image. Time-lapse movies with a larger field of view can be found in Supplementary Movies 3 and 4. **b** The sheath assembly was monitored by TssB-msfGFP localization with either *tssE* or *vasX* expressed under arabinose promoter in *tssB-msfGFP* strains lacking both *tssE and vasX*. The average number of sheaths is indicated below each image. Scale bar: 5 µm. Time-lapse movies with a larger field of view can be found in Supplementary Movie 5. Source data are provided as a Source Data file

TssE is considered to be an important baseplate component, which might anchor T6SS sheath to the baseplate^18,50^. VasX, on the other hand, is a membrane-disrupting effector secreted by T6SS in *V*. *cholerae*^51–54^. In the absence of *tssE*, T6SS activity is largely decreased as shown by live-cell imaging of TssB-msfGFP dynamics, however, not completely abolished (Fig. 5a; Supplementary Movies 3 and 4), similarly to what was shown before^17,53^. Cells lacking *vasX* assemble less T6SS during both exponential phase and early stationary phase (Fig. 5a; Supplementary Movies 3 and 4). However, in cells lacking both *tssE* and *vasX*, the T6SS assembly is abolished (Fig. 5a; Supplementary Movies 3 and 4). By contrast, cells lacking TssE and either one of effectors VgrG1, VgrG3, or TseL still assemble T6SS with a similar frequency (Supplementary Fig. 6 and Supplementary Movie 6). The ectopic expression of *tssE* from an arabinose-inducible promoter in *ΔtssE*/*ΔvasX* background restored T6SS activity to the level observed in *ΔvasX* strain, while ectopic expression of *vasX* increased frequency of T6SS assembly to the level observed in *ΔtssE* strain (Fig. 5b; Supplementary Movie 5), ruling out possible polar effects of the introduced mutations.

## Discussion

In this work, we applied a targeted proteomic approach to quantify the protein abundance of the known T6SS components from three organisms with distinct dynamics of T6SS (Fig. 2). The SID-SRM-MS method allowed us to measure the abundance of the selected components with high precision (median CV of 16.51% between peptide measurements for the same protein, median CV of 8.63% among biological triplicates). While absolute quantification of protein abundance per cell has several possible sources of error, mostly cell size variation and protein extraction and digestion efficiency, the relative comparison of protein amount and careful calibration of individual steps allow for precise determination of changes in protein abundance under various conditions. Compared to antibody-based quantification, this approach requires no specific antibody and production of recombinant proteins for calibration of immuno-detection. After initial identification of appropriate peptides, the method is fast and easily scalable to parallel analysis of tens of proteins of interest. Therefore, we believe that targeted proteomics could replace the standard immuno-detection-based approaches used to date to study regulation of T6SS assembly.

We observed a direct correlation between protein levels and their roles in the assembly and dynamics of T6SS. For instance, the membrane complex, spike, and baseplate components are known to be present in 1–12 copies per T6SS assembly^10,14^ and were indeed among the least abundant components in all three analyzed organisms. By contrast, we detected a large number of TssB/TssC and Hcp, which assemble into long polymers with more than thousand copies per single T6SS^16,18^. However, several accessory T6SS proteins are less understood and thus quantification of their abundance might help deciphering their roles in T6SS assembly. For example, activation of H1-T6SS of *P*. *aeruginosa* requires phosphorylation of Fha1 protein by inner-membrane kinase PpkA, which is presumably activated by binding of periplasmic protein TagR^31,33^. It was suggested that TagR is bound to an outer-membrane protein TagQ^32^. Interestingly, we show here that TagQ is highly abundant in *P*. *aeruginosa* and is present in about 60-fold higher copy number than TagR. This could be necessary for efficient recruitment of TagR away from PpkA to prevent futile T6SS assembly, however, TagR is itself required for PpkA activation^33^. TagS and TagT, which is predicted to be an ATP-binding component of an ABC transporter^32^, are present at about 7× lower copy number than the components of the membrane complex, suggesting that it is the enzymatic activity rather than the structure of TagST that plays an important role in T6SS activation.

Any complex biological system requires a precise and coordinated assembly of its components. The insights into mechanisms of T6SS assembly were so far mostly based on imaging of the individual components in live cells, mutagenesis, protein–protein interaction studies, and structural biology^2,55–57^. While these approaches provided critical insights, we have almost no information about how the copy number of the individual components change under various conditions and how would such changes be reflected in T6SS assembly. For example, it has been demonstrated that manipulating the level of tube protein Hcp led to the change of the length of sheath in *V*. *cholerae*, while limiting spike protein VgrG2 resulted in the reduction of the number of T6SS assemblies^53^. T6SS regulation was studied by analysis of RNA expression levels under various conditions^58–60^, however, the mRNA abundance is often not strictly correlated with protein levels for many proteins^61^, which may limit insights that such studies provide into T6SS assembly.

Here, we analyzed changes in abundance of T6SS components under conditions that significantly change frequency of T6SS assembly. Abundance of most T6SS components was changing similarly during various stages of growth of *V*. *cholerae* or when protein synthesis was inhibited by chloramphenicol. Transition from early to late exponential phase resulted in higher copy number of nearly all T6SS components, which correlated well with observed higher frequency of T6SS assembly. On the other hand, during transition to late stationary phase or during chloramphenicol treatment most components decreased in abundance but that was unlikely to explain the up to 500-fold decrease in frequency of T6SS assembly observed by live-cell imaging. Importantly, abundance of certain T6SS components varied more significantly. Most notably, the abundance of baseplate protein TssE and an effector VasX rapidly dropped under both conditions. As shown previously^53^ and here, cells lacking TssE or VasX assemble less T6SS. Interestingly, cells without both VasX and TssE assemble no T6SS (Fig. 5), however, cells lacking TssE and either one of the secreted substrates VgrG1, VgrG3, or TseL still assemble T6SS sheath with the frequency comparable to the cells lacking only TssE (Supplementary Fig. 6). This suggests that the role that VasX plays in T6SS assembly in the absence of TssE is unique because previous detailed analysis of sheath assembly in *V*. *cholerae* showed that deletion of *vgrG3* resulted in higher decrease in sheath assembly frequency than deletion of *vgrG1* or *vasX*^53^. In the absence of structural information about VasX, we can only speculate how VasX could compensate for the loss of TssE, which is predicted to be a critical component of T6SS connecting sheath to the baseplate^18^. It is possible that assembly of VasX on the VgrG spike prevents assembly of a baseplate containing TssE and that VasX itself could play a role of such a connector protein.

The rapid decrease of TssE and VasX abundance cannot be explained by protein dilution during cell growth and therefore it raises additional questions about this process. While VasX is a secreted protein, its abundance decreased faster than that of other secreted spike components, such as VgrG or PAAR. Moreover, ectopically expressed TssE or VasX also quickly disappeared from the cytosol of *E*. *coli* upon inhibition of protein synthesis. Since the amount of TssE and VasX diminished so quickly in both *V*. *cholerae* and *E*. *coli*, we speculate that these proteins evolved to be unstable and undergo proteolysis. A recent work has shown that an effector TseT secreted by H2-T6SS of *P*. *aeruginosa* requires its associated PAAR and/or chaperone protein TceT for stability, while the stability of TceT requires the co-chaperone co-TceT^62^. Interestingly, a chaperone VasW is required for secretion but not for stability of VasX^54^. It remains to be tested how TssE and VasX are degraded and what triggers their degradation. Since lack of TssE almost completely abolishes T6SS assembly, protein instability could be a general mechanism, which evolved to quickly stop T6SS activity to save energy when resources are limited, while preserving all the other T6SS components for later time.

In conclusion, our work not only provides insights into the abundance of different components of a complex bacterial nanomachine in different bacteria, but also serves as an example of a systematic study of the temporal changes of many proteins in parallel. We show that the targeted proteomics together with live-cell imaging is a powerful tool for expanding our knowledge about mechanisms behind regulation of T6SS assembly.

## Methods

### Bacterial strains and growth

A list of strains used in this work can be found in Supplementary Table 2. In general, bacterial cells were grown in Luria-Bertani (LB) media at 37 °C for *P*. *aeruginosa* and *V*. *cholerae*, and 30 °C for *A*. *baylyi* ADP1. Antibiotic concentrations used were ampicillin (300 µg/ml), streptomycin (100 µg/ml) for *V*. *cholerae,* and 50 µg/ml for *A*. *baylyi* ADP1, Irgasan (20 µg/ml), and chloramphenicol (20 µg/ml) for growth.

### Construction of mutations in *V*. *cholerae*

In-frame deletion mutants of *V*. *cholerae* were constructed by allelic exchange method using a suicide vector pWM91 as previously described^63^ in a *tssB-msfGFP* background^18^. For constructing plasmids used in complementation experiments, target genes were cloned into l-arabinose inducible vectors pBAD24 or pBAD33^64^. All cloning constructs were verified by colony PCR and sequencing. A list of oligonucleotides designed in this work can be found in Supplementary Table 3.

### Live-cell imaging

For *P*. *aeruginosa*, overnight bacterial cultures of either Δ*retS*^30^ or WT^65^ were diluted 1:50 in fresh LB medium and cultivated at 37 °C to an OD_600_ of ~1.2–1.5. For *A*. *baylyi* ADP1, overnight bacterial cultures were diluted 1:50 in fresh LB medium and cultivated at 30 °C to an OD_600_ of ~1.2–1.5. For *V*. *cholerae*, overnight bacterial cultures were diluted 1:200 in fresh LB medium and cultivated to desired OD_600_. For the complementation experiments from pBAD vectors, the expression was induced by diluting overnight cultures 1:250 in fresh LB medium supplemented with antibiotics, as well as 0.03% l-arabinose and cultivated at 37 °C to an OD_600_ of ~1.2–1.5. One milliliter of cells were concentrated 10 times before spotted onto a thin pad containing 1% LB and covered with a coverslip. Bacterial cells were imaged as described^47,53^. The Nikon Ti-E inverted motorized microscope used in this work was equipped with Perfect Focus System and Plan Apo 1003 Oil Ph3 DM (NA 1.4) objective lens, SPECTRA X light engine (Lumencor), along with ET-ECFP (Chroma #49001), ET-GFP (Chroma #49002), and ET-mCherry (Chroma #49008) filter set for fluorescence excitation and filtration. sCMOS camera pco.edge 4.2 with pixel size of 65 nm (PCO) and VisiView software (Visitron Systems) were used for imaging. Humidity was set to 95% regulated by Okolab T-unit (Okolab). Imaging for different bacteria was set at the desired growth temperature. The duration and intervals of imaging were indicated in figures. The image analysis was conducted in Fiji^66^ and customized plugin based on StackReg^67^. The quantification of sheath number in the cell was conducted in Fiji. The total cell number was accessed from the phase contrast image using “Find Maxima” with “edge maxima exclusion” function activated. The noise tolerance setting was adjusted manually for each case. The number of sheath was counted from the corresponding GFP image using “Find Maxima” with “edge maxima exclusion” function activated. The noise tolerance setting was adjusted manually for each case. At least 1000 cells were analyzed for each strain.

### Time course of *V*. *cholerae* growth

Overnight bacterial cultures of strain B625 (*V*. *cholerae tssB-msfGFP*) were diluted 1:200 in fresh LB medium and cultivated to different OD_600_ as indicated in the results. For each time point, ~10^9^ cells were harvested for the sample preparation for mass spectrometry, and another 1 ml of cells suspension was concentrated for live-cell imaging. Three independent replicates were analyzed.

### Chloramphenicol treatment of *V*. *cholerae*

Overnight bacterial cultures of strain B625 (*V*. *cholerae tssB-msfGFP*) were diluted 1:200 in fresh LB medium and cultivated to an OD_600_ of ~1.2–1.5. Chloramphenicol was added to the cultures to a final concentration of 1 mg/ml. 0, 20, 45, and 90 min after the treatment, ~10^9^ cells were harvested for the sample preparation for mass spectrometry, with another 1 ml of cells were concentrated for live-cell imaging. Three independent replicates were performed.

### Chloramphenicol treatment of *E*. *coli*

Overnight bacterial cultures of strains B348 (*E*. *coli pBAD24-tssE*) or LLB397 (*E*. *coli pBAD24-vasX*) were diluted 1:100 in fresh LB medium supplemented with ampicillin and 0.003% and 0.03% of arabinose for induction, respectively. Cultures were then cultivated to an OD_600_ of ~1.2–1.5. Chloramphenicol was added to the cultures to a final concentration of 1 mg/ml. 0 and 90 min after the treatment, ~10^9^ cells were harvested for the sample preparation for mass spectrometry. Three independent replicates were analyzed.

### Sample preparation for mass spectrometry

Bacterial cultures grown at desired OD_600_ were prepared as described for live-cell imaging. Pellets of ~10^9^ cells were washed three times with PBS at 4 °C and 8000 × *g* for 10 min in a table top centrifuge. Cell pellets were then dissolved in lysis buffer (1% sodium-deoxycholate (SOC) in 100 mM ammoniumbicarbonate) followed by the addition of 10 mM tris‐2‐carboxyethyl‐phosphine (TCEP) to enable good extraction of proteins from bacterial cells^43^. Samples were sonicated using VialTweeter (Hielscher), followed by an incubation at 95 °C for 10 min. Samples were then subjected to the sonication with 30 s on and 30 s off for 10–20 cycles using Bioruptor (Diagenode) until samples became clear. After the complete lysis, the protein content was measured by BCA assay for each sample (Pierce, Fisher Scientific). 15 mM of chloroacetamide (Sigma) was added to the sample and incubated at 25 °C for 30 min with gentle agitation. Lysyl-endopeptidase (Wako Diagnostics) was added to lysates of 100 μg of proteins in a final enzyme/protein ratio of 1:200 (w/w). The mixtures were then incubated at 37 °C for 4 h. Peptides were then subjected to the digestion by trypsin in a final enzyme/protein ratio of 1:50 (w/w) for 12 h at 37 °C. 5% of trifluoroacetic acid was added to a final concentration of 1%. The precipitates were removed by centrifugation while the supernatants were subjected to C18 solid phase extraction using Macrospin columns (Harvard Apparatus). The eluted peptides were dried at 55 °C under vacuum and then resuspended in a buffer containing 0.15% formic acid and 2% acetonitrile. The resuspensions were fully dissolved by using 10 s of ultrasonication and 5 min incubation at 25 °C. Two C-terminally stable isotope-labeled proteotypic peptide mixtures (SpikeMix L, JPT peptides) were added to each sample. For each protein of interest, up to five peptides were included in the pools. To select stable isotope-labeled proteotypic peptide suitable for the SRM, the whole-cell preparation from each organism were analyzed by shotgun proteomics first. Peptides from the whole-cell samples were prepared as described above and cleaned up using SP3 protocol with minor modification^68^. Only peptides that were unique for proteins of interest and detected significantly by shotgun proteomic LC–MS analysis were included for the synthesis. In general, for each sample, 15 μl of peptides from biological samples containing ~7.5 μg of peptides were mixed with 5 μl of ~0.2 pmol of SpikeMix (the exact concentration of each isotope-labeled peptide was determined as described below). The list of peptides included in both SpikeMix L mixtures employed can be found in Supplementary Data 4. The peptides in these mixtures were isotopically labeled at the C-terminus with either heavy lysine or arginine acid. Both SpikeMix mixtures were dissolved in a buffer containing 20% acetonitrile and 0.1% of trifluoroacetic acid. The samples were finally subjected to SRM analysis in a TSQ Vantage mass spectrometer (Thermo Scientific). Each sample was prepared and analyzed in biological triplicate. To avoid peptide losses through hydrophobic interactions with plastic surfaces, only low binding tips, tubes or glass vials were used when handling peptide standard solutions.

### Cell enumeration by FACS

The Bacteria Counting Kit (Thermo Fischer) was used to enumerate bacteria by flow cytometry according to manufacturer protocol with minor modification. Cells grown at desired OD_600_ were prepared as described above and diluted with filter-sterilized 5% LB, 0.15 M NaCl to a final density of ~10^6^ cells/ml for further analysis. For each 1 ml of sample, 1 μl of SYTO BC bacteria stain was added and incubated at room temperature in the dark for at least 5 min. Meanwhile, the microsphere standard suspension containing 10^8^ beads/ml was fully resuspended and sonicated in a water bath for 5 min. Ten microliters of such suspension was added to the stained cells to have a final concentration of 10^6^ beads/ml. The stained mixtures were subjected to the analysis in a flow cytometer (BD Bioscience) equipped with ex488-LP502-BP530/30 laser. The estimation of cells number in the 1 ml sample was as following: The number of cells/1 OD_600_/ml = the number of counting in the bacteria frame/the number of counting in the microsphere frame × 10^6^ × dilution factor/measured OD_600_ value. Three replicates were performed for each condition, for example data and our gating strategy (see Supplementary Fig. 8).

The protein content per cell for each bacterial species was estimated as following: a complete extraction of protein contents from known number of cells for each bacteria was performed as described above. The total protein contents were measured by BCA assay (Pierce, Fisher Scientific). The total protein content divided by the cell number provides the protein content per each cell. Three replicates were performed. For all SRM analyses, the number of cells where native peptides were extracted from was estimated using the following formula: number of cells = peptide concentration × volume of sample/protein content per cell.

### Determination of peptide concentration in SpikeMix

In total, 130 peptides that displayed good qualities from SRM measurement were selected for further synthesis of quantified, proteotypic peptides that have a C-terminal tag that can be cleaved by tryptic digestion (SpikeTides™_TQ; the list can be found in Supplementary Data 5). One hundred and twenty-nine out of them could be synthesized with the desired quality. The synthesized peptides were then dissolved to a final concentration of 10 pmol/μl) in a buffer containing 20% acetonitrile and 0.1% of trifluoroacetic acid. 100 pmol per quantified peptide were mixed and dried under a vacuum at 55 °C. The dried peptide mixture was then dissolved in a buffer containing 1 M urea and 0.1 M ammoniumbicarbonate, and subjected to ultrasonication for 10 s, followed by 10 min incubation at 25 °C. The mixture was then digested with trypsin for 12 h at 37 °C. The digested product was then acidified with a final concentration of 1% trifluoroacetic acid. The two isotope-labeled peptide mixtures with ~100 pmol per each peptide were mixed with the digested light quantified peptides and subjected to C18 solid phase extraction. After elution, the peptides were dried under vacuum at 55 °C. The dried mixtures were then dissolved in a buffer containing 0.15% formic acid and 2% acetonitrile. The resuspensions were fully dissolved by using 10 s of ultrasonication and 5-min incubation at 25 °C. The samples were diluted with 0.1% formic acid to a final concentration of 250 fmol/µl/peptide. 2 µl of this solution was injected into the LC–MS instrument for SID-SRM-MS analysis. To avoid peptide losses through hydrophobic interactions with plastic surfaces, only low binding tips, tubes, or glass vials were used when handling peptide standard solutions. The samples were subjected to SID-SRM-MS analysis to determine the precise concentrations of all selected heavy peptides. All samples were prepared and analyzed in triplicates.

### SRM LC–MS analysis

In the first step, SRM assays^69^ were generated from a mixture containing ~500 fmol of each heavy reference peptide (mixture of both SpikeMix L mixes), as well as iRT peptides^70^ and shotgun LC–MS/MS analysis on a Q-Exactive HF platform. The setup of the μRPLC–MS system was as described previously^71^. Chromatographic separation of peptides was carried out using an EASY nano-LC 1000 system (Thermo Fisher Scientific), equipped with a heated RP-HPLC column (75 μm ×  37 cm) packed in-house with 1.9 μm C18 resin (Reprosil-AQ Pur, Dr. Maisch). Peptides were analyzed per LC–MS/MS run using a linear gradient ranging from 95% solvent A (0.1% formic acid) and 5% solvent B (99.9% acetonitrile, 0.1% formic acid) to 45% solvent B over 60 min at a flow rate of 200 nl/min. Mass spectrometry analysis was performed on Q-Exactive HF mass spectrometer equipped with a nanoelectrospray ion source (both Thermo Fisher Scientific). Each MS1 scan was followed by high-collision-dissociation (HCD) of the 10 most abundant precursor ions with dynamic exclusion for 20 s. Total cycle time was ~1 s. For MS1, 3e6 ions were accumulated in the Orbitrap cell over a maximum time of 100 ms and scanned at a resolution of 120,000 FWHM (at 200*m*/*z*). MS2 scans were acquired at a target setting of 1e5 ions, accumulation time of 50 ms and a resolution of 30,000 FWHM (at 200*m*/*z*). Singly charged ions and ions with unassigned charge state were excluded from triggering MS2 events. The normalized collision energy was set to 27%, the mass isolation window was set to 1.4*m*/*z* and one microscan was acquired for each spectrum. The acquired raw-files were searched against a decoy database using the MaxQuant software (Version 1.0.13.13) containing normal and reverse sequences of the predicted UniProt entries of *V. cholerae* serotype O1 (strain ATCC 39315/El Tor Inaba N16961), *P. aeruginosa* (strain ATCC 15692/DSM 22644/CIP 104116/JCM 14847/LMG 12228/1C/PRS 101/PAO1), and *A. baylyi* (strain ATCC 33305/BD413/ADP1) (www.ebi.ac.uk, release date 2016/07/19), retention time standard peptides and commonly observed contaminants (in total 25,934 sequences) generated using the SequenceReverser tool from the MaxQuant software (Version 1.0.13.13). The precursor ion tolerance was set to 10 ppm and fragment ion tolerance was set to 0.02 Da. The search criteria were set as follows: full tryptic specificity was required (cleavage after lysine or arginine residues unless followed by proline), three missed cleavages were allowed, carbamidomethylation (C) was set as fixed modification. Heavy-labeled arginine (+10 Da − ^13^C(6)^15^N(4)), heavy-labeled lysine (+8 Da − ^13^C(6)^15^N(2)) and oxidation (*M*) were set as a variable modifications. The resulting msms.txt file was converted to a spectral library panel with the 5–10 best transitions for each peptide using an in-house software tool. This was then imported into the SpectroDive program (Version 8.0, Biognosys, Schlieren, Switzerland) and a scheduled transition list for quantitative SRM analysis was generated. Here, all samples were analyzed on a TSQ-Vantage triple-quadrupole mass spectrometer coupled to an Easy-nLC (Thermo Fisher, Scientific). In each injection an equivalent of 1.5 μg of peptides including heavy peptide references was loaded onto a custom-made main column (Reprosil C18 AQ, 3 μm diameter, 100 Å pore, 0.75 × 300 mm) and separated using the same gradient mentioned above. The mass spectrometer was operated in the positive ion mode using ESI with a capillary temperature of 275 °C, a spray voltage of +2200 V. All of the measurements were performed in an unscheduled mode and a cycle time of 2 s. A 0.7 FWHM resolution window for both Q1 and Q3 was set for parent-ion and product-ion isolation. Fragmentation of parent-ions was performed in Q2 at 1.2 mTorr, using collision energies calculated with the SpectroDive software (version 8.0). Each condition was analyzed in biological triplicates. All raw-files were imported into SpectroDive for absolute peptide and protein quantification. Here, only elution groups with *q*-values < 0.01 were considered correct calls and used for quantification. Median heavy/light ratios of triplicates were employed to determine protein levels. The absolute abundance of each protein determined by each peptide was calculated as following: target-to-reference ratio × spiked in heavy peptide amount × Avogadro’s number/cell number. Significance of different peptide abundances under different conditions was calculated using *p*-values from Tukey’s multiple comparison test in Prism GraphPad 7.04. For the time-course experiment, such tests were performed on the copy number of each protein under different conditions. For the protein synthesis inhibition experiment, such test was performed on the relative levels to TssB for each protein following the treatment. It should be noted that the quantification of VasX was performed using crude peptides, since no quantified, proteotypic peptide was available for determining the precise concentration of the heavy peptides used for VasX.

### Reporting summary

Further information on research design is available in the Nature Research Reporting Summary linked to this article.

## Supplementary information

Supplementary Information

Description of Additional Supplementary Files

Supplementary Data 1

Supplementary Data 2

Supplementary Data 3

Supplementary Data 4

Supplementary Data 5

Supplementary Data 6

Supplementary Data 7

Supplementary Movie 1

Supplementary Movie 2

Supplementary Movie 3

Supplementary Movie 4

Supplementary Movie 5

Supplementary Movie 6

Reporting Summary

Source Data

## Data Availability

The mass spectrometry proteomics data have been deposited to the ProteomeXchange Consortium^72^ (http://proteomecentral.proteomexchange.org) via the PRIDE partner repository^73^ with the dataset identifier PXD012832 [https://www.ebi.ac.uk/pride/archive/projects/PXD012832]. The source data for Figs. 2b–d, 3a, b, 4a, b, and 5a, b and Supplementary Figs. 1, 2, 3, 4a, b, and 5 are provided as a Source Data file. A reporting summary for this Article is available as a Supplementary Information file. All other data supporting the findings of this study are available from the corresponding author on reasonable request.
