# Supplementary material for: Abundance of bacterial Type VI secretion system components measured by targeted proteomics
Source: Nat Commun. 2019 Jun 13;10:2584. doi: 10.1038/s41467-019-10466-9 (PMC6565705; doi:10.1038/s41467-019-10466-9)
Supplement: Supplementary file 1 — Supplementary Information [file 41467_2019_10466_MOESM1_ESM.pdf]

Abundance of bacterial Type VI secretion system components measured by targeted proteomics.

Lin et al.

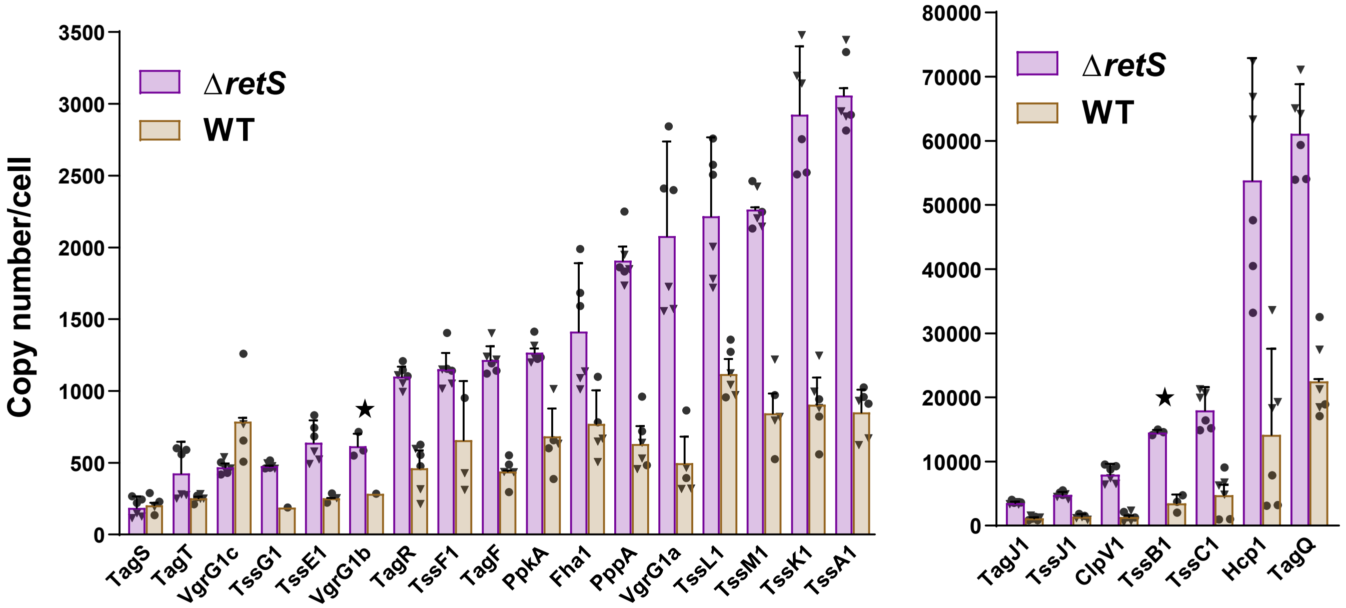

Supplementary Figure 1. The abundance of T6SS components in *P. aeruginosa* in the presence or absence of RetS. The absolute copy number of each selected component of H1-T6SS in whole cell lysate of *P. aeruginosa* PAO1 (*ΔretS* background or WT background) grown to OD_{600} ~1.5. Bar graph shows the average of copy number determined by two peptides (individual measurements shown with dots for peptide 1 or triangles for peptide 2), with error bar as the SD between measurements from both peptides if available; otherwise, SD between biological replicates is shown (highlighted with a star). Note the *y*-axis is shown in linear scale. The detailed quantification for this figure can be found in Supplementary Data 1&6. Source data are provided as a Source Data file.

Supplementary Figures

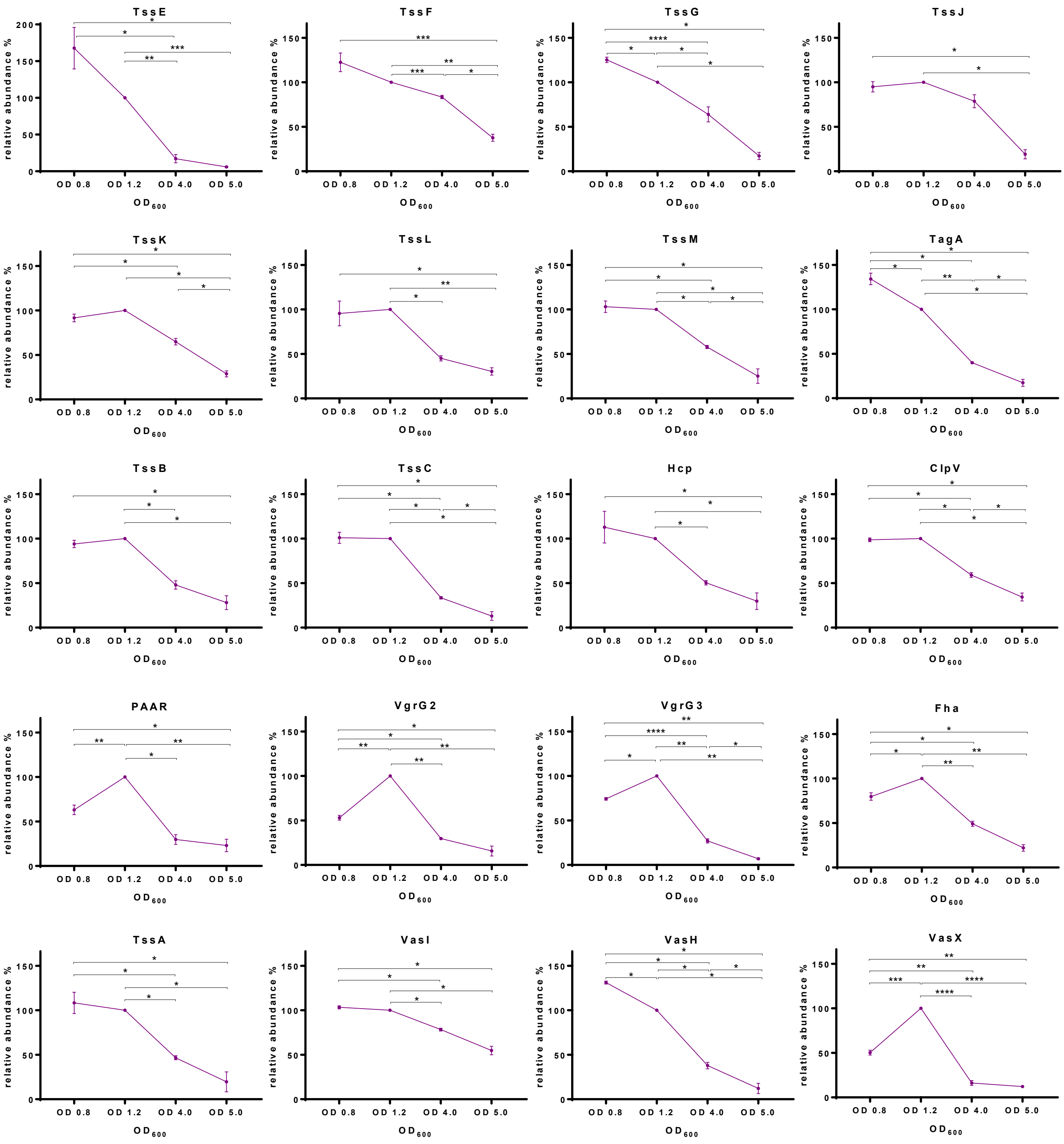

Supplementary Figure 2. The change in abundance of *V. cholerae* T6SS proteins during growth phases. The relative abundance levels of each component during 4 growth phases (OD_{600} ~0.7, ~1.2, ~4, and ~5, respectively). Note the *y*-axis is shown as % abundance related to the protein levels at T2 (OD_{600} ~1.2). The error bar represents the standard deviation of measurements from three replicates. For statistical analysis, the one-way ANOVA with multiple comparison using Tukey correction was performed. *: $p \leq 0.05$, **: $p \leq 0.01$, ***: $p \leq 0.001$, ****: $p \leq 0.0001$. The detailed quantification for this figure can be found in Supplementary Data 2. Source data are provided as a Source Data file.

Supplementary Figures

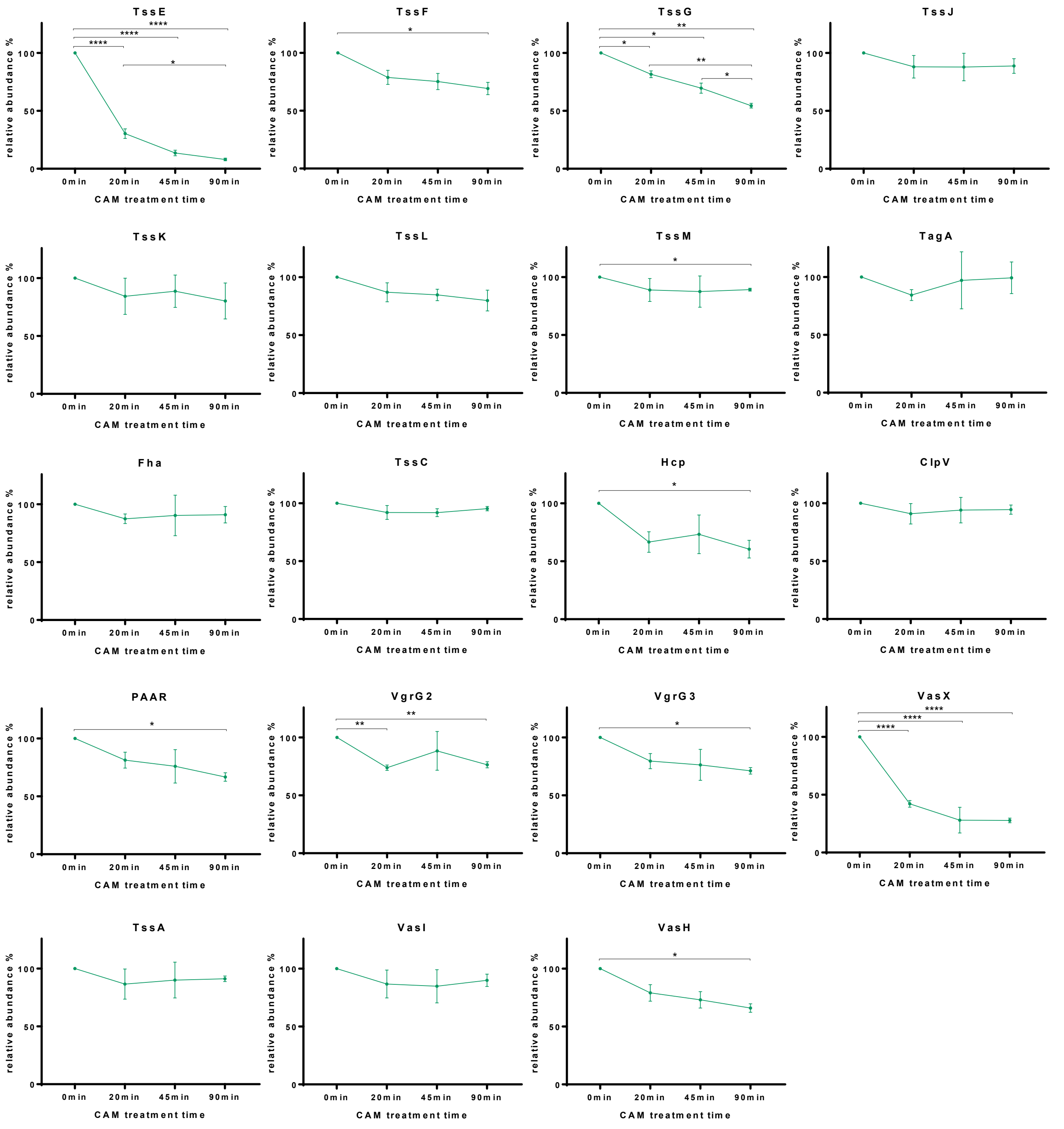

Supplementary Figure 3. The change of abundance of *V. cholerae* T6SS components in response to chloramphenicol treatment. The relative abundance levels of each component at 4 time points following the CAM treatment (0 min, 20 min, 45 min, and 90 min, respectively). Note the *y*-axis is shown as % abundance normalized to TssB related to the protein levels at T0 (0 min; pre-treatment). The error bar represents the standard deviation of measurements from three replicates. For statistical analysis, the one-way ANOVA with multiple comparison using Tukey correction was performed. *: $p \leq 0.05$, **: $p \leq 0.01$, ***: $p \leq 0.001$, ****: $p \leq 0.0001$. The detailed quantification for this figure can be found in Supplementary Data 3. Source data are provided as a Source Data file.

Supplementary Figures

A

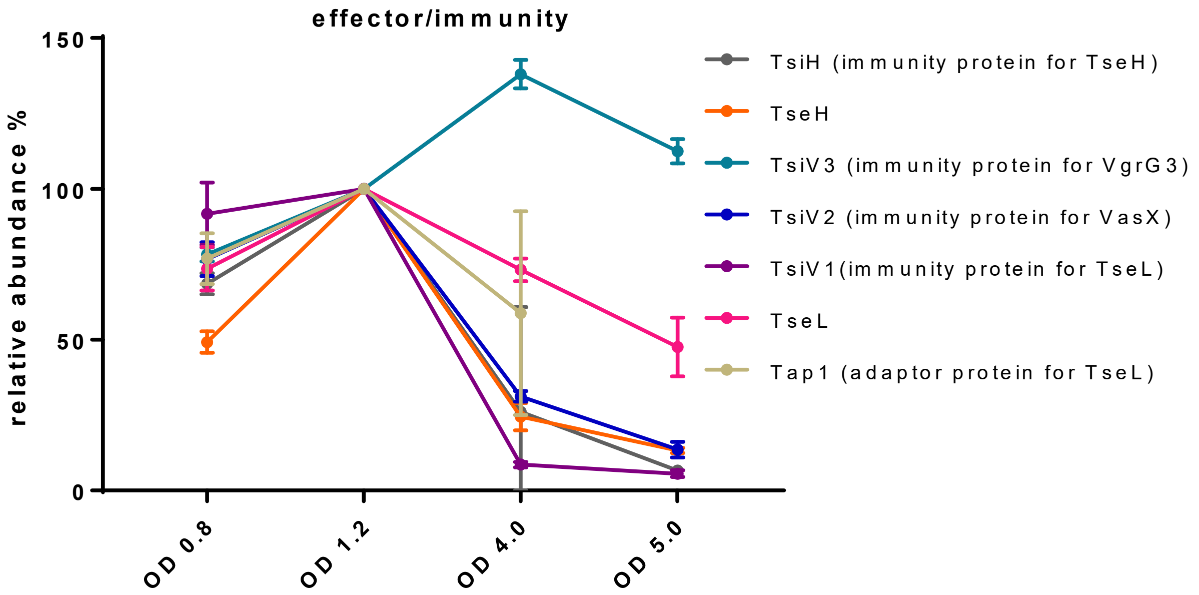

B

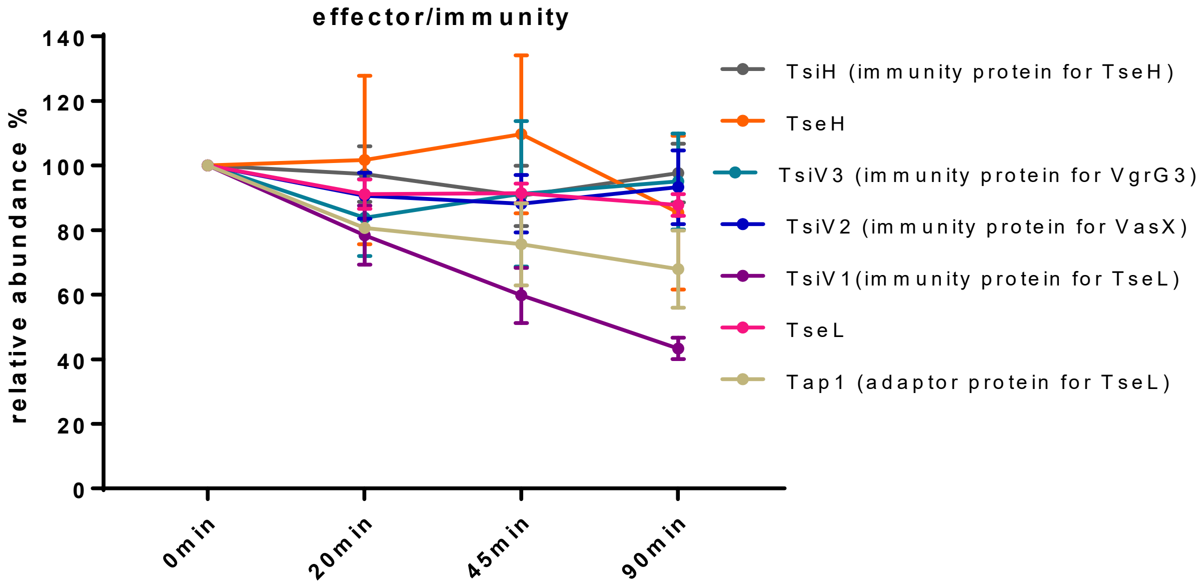

Supplementary Figure 4. The change in abundance of *V. cholerae* T6SS effector and immunity proteins under different conditions. (A) The relative abundance levels of each component during 4 growth phases (OD_{600} ~0.7, ~1.2, ~4, and ~5, respectively). Note the *y*-axis is shown as % abundance related to the protein levels at T2 (OD_{600} ~1.2). The error bar represents the standard deviation of measurements from three replicates. (B) The relative abundance levels of each component at 4 time points following the CAM treatment (0 min, 20 min, 45 min, and 90 min, respectively). Note the *y*-axis is shown as % abundance normalized to TssB related to the protein levels at T0 (0 min; pre-treatment). The error bar represents the standard deviation of measurements from three replicates. Source data are provided as a Source Data file.

Supplementary Figures

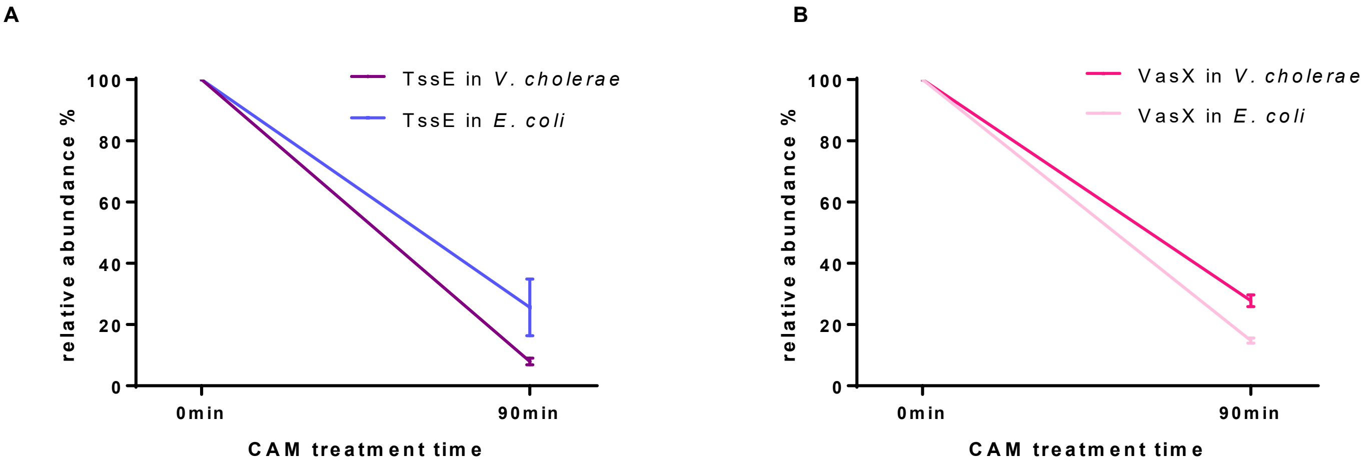

Supplementary Figure 5. The change of abundance of TssE and VasX when overproduced in *E. coli* in response to chloramphenicol treatment. (A) The relative abundance levels of TssE in *E. coli* or *V. cholerae* following the CAM treatment (0 min and 90 min). (B) The relative abundance levels of VasX in *E. coli* or *V. cholerae* following the CAM treatment (0 min and 90 min). Note the *y*-axis is shown as % abundance normalized to the protein levels at T0 (0 min; pre-treatment). The error bar represents the standard deviation of measurements from three replicates. The detailed quantification for this figure can be found in Supplementary Data 7. Source data are provided as a Source Data file.

Supplementary Figures

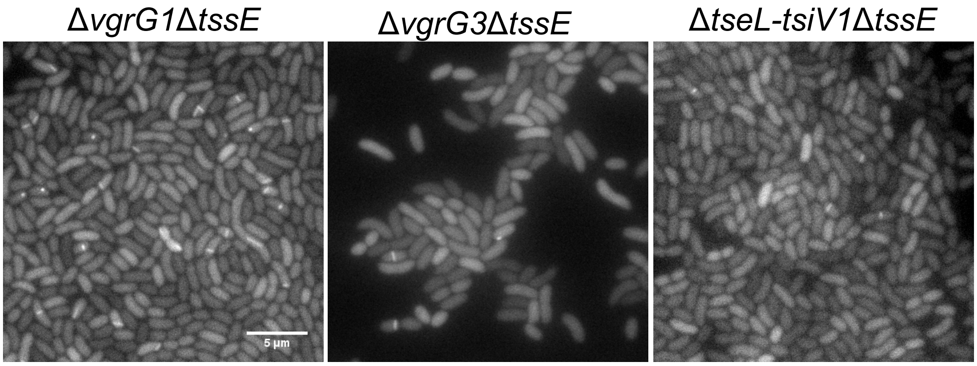

Supplementary Figure 6. *V.cholerae* lacking TssE and one of the other effector proteins harbors residual T6SS assembly. The sheath assembly was monitored by TssB-msfGFP localization in *ΔtssE ΔvgrG1*, *ΔtssE ΔvgrG3* or *ΔtssE ΔtseL-tsiV1* cells. Representative images of GFP channel are shown for each indicated strain (all *tssB-msfGFP* background). Scale bar: 5 µm. Time-lapse movies with a larger field of view can be found in Supplementary Movie 6.

Supplementary Figures

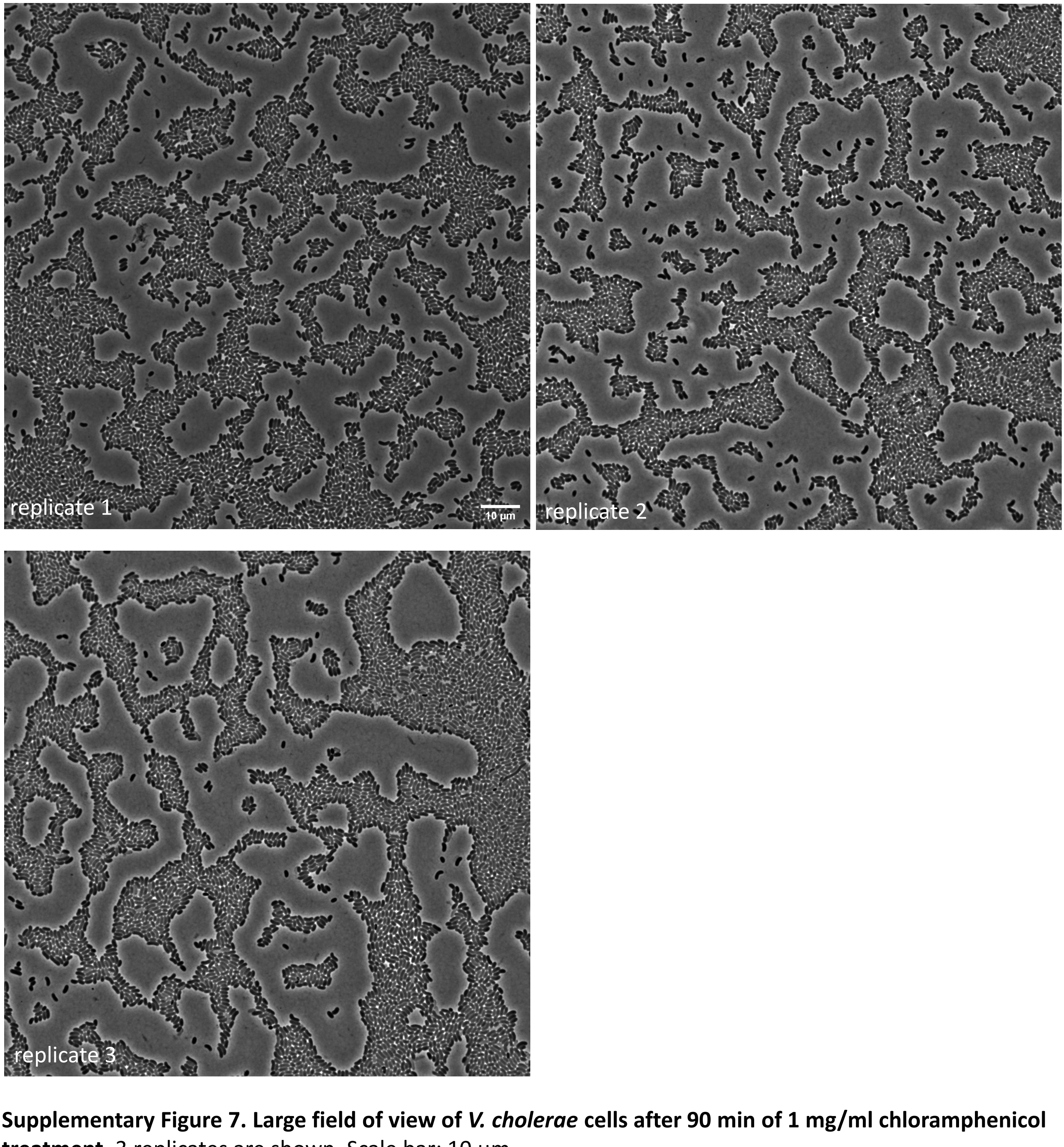

Supplementary Figure 7. Large field of view of *V. cholerae* cells after 90 min of 1 mg/ml chloramphenicol treatment. 3 replicates are shown. Scale bar: 10 µm.

Supplementary Figures

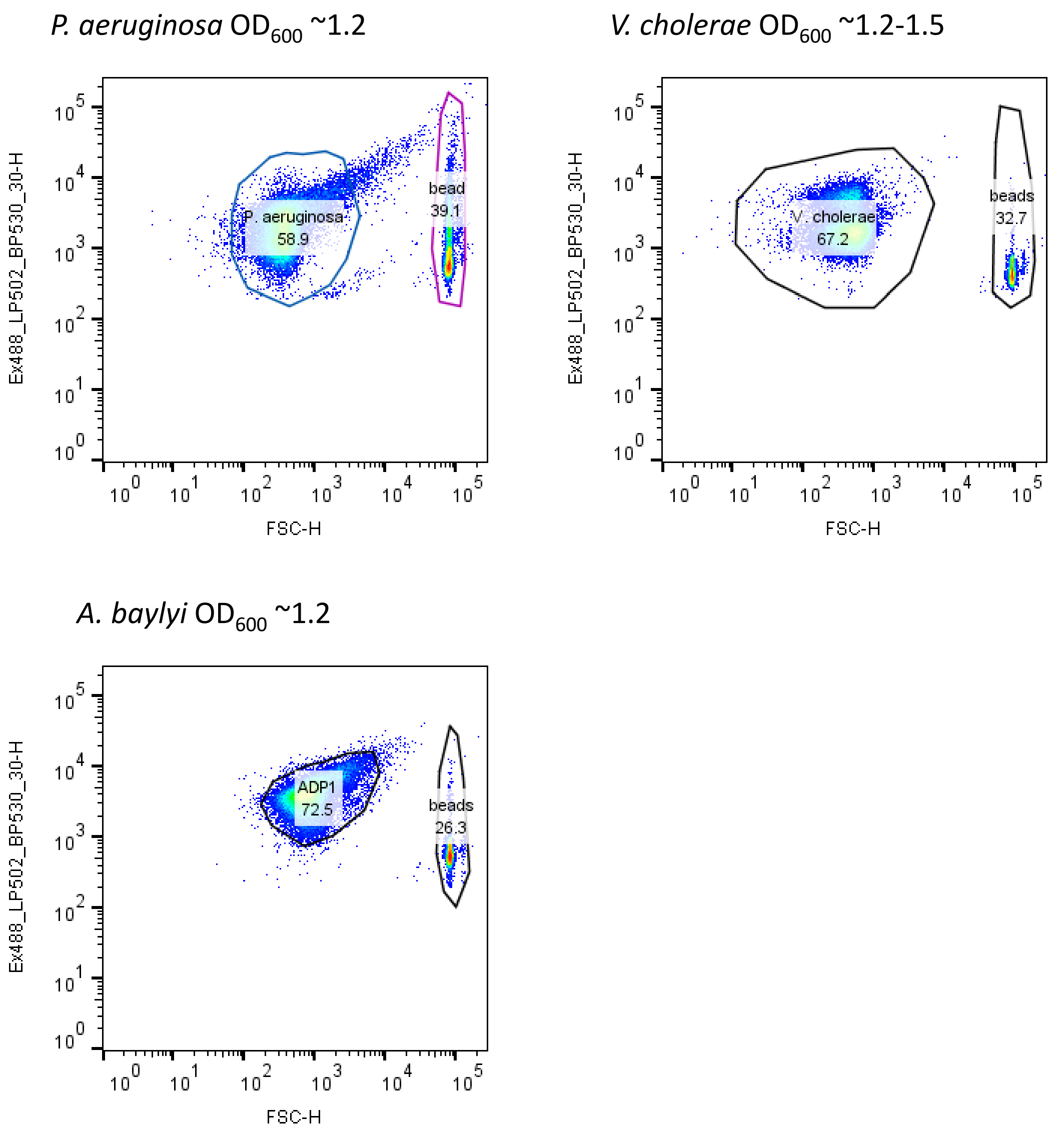

Supplementary Figure 8. Flow cytometry gating strategy for bacterial cell counting. One example plot per bacterial strain is shown. 1 ml of sample containing ~10^6 cells was stained with SYTO BC bacterial DNA stain and then mixed with 10 µl of microsphere standard beads. The stained mixtures were subjected to the analysis in a flow cytometer (BD Bioscience) equipped with ex488-LP502-BP530/30 laser (green).

Supplementary Tables

Supplementary Table 1. Cell enumeration by FACS for *P. aeruginosa, V. cholerae* and *A. baylyi*.

strain	OD_{600}	cell number/1ml OD_{600} 1	CV for cell number estimation	fg/cell	CV for fg/cell estimation
P. aeruginosa PAO1 *ΔretS*	~1.2	5.47×10^8	25.58	278.48	5.02
V. cholerae 2740-80	~0.5	4.44×10^8	12.75	259.06	5.36
V. cholerae 2740-80	~1.5	1.00×10^9	13.90	182.21	7.45
V. cholerae 2740-80	~4.0	1.77×10^9	13.39	156.39	9.08
A. baylyi ADP1	~1.2	8.73×10^8	7.46	208.60	7.34

Supplementary Table 2. Strains and plasmids used in this work.

**Bacterial strains**	**Genotypes**	**Plasmids**	**References**
B131	*P. aeruginosa ΔretS*		(Mougous et al., 2006)[1]
B133	*P. aeruginosa*		(Mougous et al., 2006; Stover et al., 2000)[1,2]
B274	V. cholerae 2740-80		(Basler et al., 2012)[3]
B278	A. baylyi ADP1, rpsL'-K88R		(Basler et al., 2013)[4]
B475	*P. aeruginosa ΔretS clpV-sfGFP*		(Vettiger et al., 2016)[5]
B625	*V. cholerae 2740-80, tssB-msfGFP*		(Kudryashev et al. 2015)[6]
DH022	*A. baylyi ADP1, rpsL'-K88R, tssB-sfGFP, clpV-mCherry*		(Ringel et al., 2017)[7]
B630	*V. cholerae 2740-80, tssB-msfGFP, ΔtssE*		(Vettiger et al., 2016)[5]
AV029	*V. cholerae 2740-80, tssB-msfGFP, ΔvgrG3*		(Vettiger et al., 2016)[5]
AV034	*E. coli SM10 λpir*	pWM91-ΔvasX	(Vettiger et al., 2016)[5]
B294	*E. coli SM10 λpir*	pWM91-ΔtssE	(Vettiger et al., 2016)[5]
AV041	*E. coli SM10 λpir*	pWM91-ΔvgrG1	(Vettiger et al., 2016)[5]
AV060	*E. coli SM10 λpir*	pWM91-ΔtseL-tsiV1	(Vettiger et al., 2016)[5]
LLB382	*V. cholerae 2740-80, tssB-msfGFP, ΔtssE ΔvasX*		this work
LLB386	*V. cholerae 2740-80, tssB-msfGFP, ΔvasX*		this work
LLB390	*V. cholerae 2740-80, tssB-msfGFP, ΔtssE ΔvasX*	*pBAD33-tssE*	this work
LLB402	*V. cholerae 2740-80, tssB-msfGFP, ΔtssE ΔvasX*	*pBAD24-vasX*	this work
B348	*E. coli DH5α λpir*	*pBAD24-tssE*	this work
XB029	*E. coli DH5α λpir*	*pBAD33-tssE*	this work
LLB397	*E. coli DH5α λpir*	*pBAD24-vasX*	this work
LLB442	*V. cholerae 2740-80, tssB-msfGFP, ΔtssE ΔvgrG1*		this work
LLB443	*V. cholerae 2740-80, tssB-msfGFP, ΔtssE ΔtseL-tsiV1*		this work
LLB383	*V. cholerae 2740-80, tssB-msfGFP, ΔtssE ΔvgrG3*		this work

Supplementary Tables

Supplementary Table 3. primers used in this work.

oligos	seqeunce 5'-3'
vasX-SpeI-for	TCAGTAACTAGTatgagtaatcccaatcaagc
vasX-XmaI-rev	TCAGTACCCGGGttaaccttttcctacaacga
tssE_SpeI_For	TCAGTAACTAGTATGACGTACATCGCACCTGA
tssE-XmaI-rev	TCAGTACCCGGGTTAAAACACTCGATATTTTCTGC
tssE_pBAD33_For	TCAGTAGCTAGCAGGAGGAATTCACCATGACGTACATCGCACCTGAAG

Supplementary References

1. Mougous JD*, et al.* A virulence locus of Pseudomonas aeruginosa encodes a protein secretion apparatus. *Science* **312**, 1526-1530 (2006).
2. Stover CK*, et al.* Complete genome sequence of Pseudomonas aeruginosa PAO1, an opportunistic pathogen. *Nature* **406**, 959-964 (2000).
3. Basler M, Mekalanos JJ. Type 6 secretion dynamics within and between bacterial cells. *Science* **337**, 815 (2012).
4. Basler M, Ho BT, Mekalanos JJ. Tit-for-tat: type VI secretion system counterattack during bacterial cell-cell interactions. *Cell* **152**, 884-894 (2013).
5. Vettiger A, Basler M. Type VI Secretion System Substrates Are Transferred and Reused among Sister Cells. *Cell* **167**, 99-110 e112 (2016).
6. Kudryashev M*, et al.* Structure of the type VI secretion system contractile sheath. *Cell* **160**, 952-962 (2015).
7. Ringel PD, Hu D, Basler M. The Role of Type VI Secretion System Effectors in Target Cell Lysis and Subsequent Horizontal Gene Transfer. *Cell Rep* **21**, 3927-3940 (2017).
