# Supplementary material for: Abundance of bacterial Type VI secretion system components measured by targeted proteomics
Source: Nat Commun. 2019 Jun 13;10:2584. doi: 10.1038/s41467-019-10466-9 (PMC6565705; doi:10.1038/s41467-019-10466-9)
Supplement: Supplementary file 2 — Description of Additional Supplementary Files [file 41467_2019_10466_MOESM2_ESM.docx]

**Description of Supplementary Files**

**File Name:** **Supplementary Data 1.**

**Description:** The detailed quantification of copy number per cell for T6SS components, related to Figure 2.

**File Name: Supplementary Data 2.**

**Description:** The detailed quantification of abundance for T6SS components in *V. cholerae* during different growth phases, related to Figure 3.

**File Name:** **Supplementary Data 3.**

**Description:** The detailed quantification of abundance for T6SS components in *V. cholerae* following the chloramphenicol treatment, related to Figure 4.

**File Name:** **Supplementary Data 4.**

**Description:** The list of peptides in the stable isotope-labeled peptide mixtures.

**File Name:** **Supplementary Data 5.**

**Description:** The list of quantified, proteotypic peptides that have a C-terminal tag that can be cleaved by tryptic digestion (SpikeTides ™_TQ).

**File Name:** **Supplementary Data 6.**

**Description:** The detailed quantification of copy number per cell for T6SS components in *P. aeruginosa* PAO1 WT. Related to Supplementary Figure 1.

**File Name:** **Supplementary Data 7.**

**Description:** The detailed quantification of abundance for TssE or VasX in *E. coli* following the chloramphenicol treatment, related to Supplementary Figure 5.

**File Name:** **Supplementary Movie 1.**

**Description:** The sheath assembly monitored by TssB-msfGFP localization in *V. cholerae* cells during different growth phases, related to Figure 3A. Time-lapse series were acquired during 5 min with a 10 s interval. Videos play at a rate of 7 frame/s. Field of view for each movie is 52 × 52 μm. Scale bar: 5 µm.

**File Name:** **Supplementary Movie 2.**

**Description:** The sheath assembly monitored by TssB-msfGFP localization in *V. cholerae* cells after 1 mg/ml of CAM treatment, related to Figure 4A. Time-lapse series were acquired during 5 min with a 10 s interval. Videos play at a rate of 7 frame/s. Field of view for each movie is 52 × 52 μm. Scale bar: 5 µm.

**File Name:** **Supplementary Movie 3.**

**Description:** The sheath assembly monitored by TssB-msfGFP localization in *V. cholerae* cells in the absence of TssE, VasX or both at OD600 ~0.6, related to Figure 5A. Time-lapse series were acquired during 5 min with a 10 s interval. Videos play at a rate of 7 frame/s. Field of view for each movie is 52 × 52 μm. Scale bar: 5 µm.

**File Name:** **Supplementary Movie 4.**

**Description:** The sheath assembly monitored by TssB-msfGFP localization in *V. cholerae* cells in the absence of TssE, VasX or both at OD600 ~1.5, related to Figure 5A. Time-lapse series were acquired during 5 min with a 10 s interval. Videos play at a rate of 7 frame/s. Field of view for each movie is 52 × 52 μm. Scale bar: 5 µm.

**File Name:** **Supplementary Movie 5.**

**Description:** The sheath assembly monitored by TssB-msfGFP localization in *V. cholerae* cells with either *tssE* or *vasX* expressed under arabinose promoter in TssB-msfGFP strains lacking both *tssE* and *vasX*, related to Figure 5B. Time-lapse series were acquired during 5 min with a 10 s interval. Videos play at a rate of 7 frame/s. Field of view for each movie is 52 × 52 μm. Scale bar: 5 µm.

**File Name:** **Supplementary Movie 6.**

**Description:** The sheath assembly was monitored by TssB-msfGFP localization in *ΔtssE ΔvgrG1*, *ΔtssE ΔvgrG3* or *ΔtssE ΔtseL-tsiV1* cells at OD600 ~1.5, related to Supplementary Figure 6. Time-lapse series were acquired during 5 min with a 10 s interval. Videos play at a rate of 7 frame/s. Field of view for each movie is 52 × 52 μm. Scale bar: 5 µm.
