# Supplementary material for: Abundance of bacterial Type VI secretion system components measured by targeted proteomics
Source: Nat Commun. 2019 Jun 13;10:2584. doi: 10.1038/s41467-019-10466-9 (PMC6565705; doi:10.1038/s41467-019-10466-9)
Supplement: Supplementary file 16 — Reporting Summary [file 41467_2019_10466_MOESM16_ESM.pdf]

nature research

Corresponding author(s): Marek Basler

Last updated by author(s): May 9, 2019

Reporting Summary

Nature Research wishes to improve the reproducibility of the work that we publish. This form provides structure for consistency and transparency in reporting. For further information on Nature Research policies, see Authors & Referees and the Editorial Policy Checklist.

Statistics

For all statistical analyses, confirm that the following items are present in the figure legend, table legend, main text, or Methods section.

n/a	Confirmed	
☐	☒	The exact sample size (n) for each experimental group/condition, given as a discrete number and unit of measurement
☐	☒	A statement on whether measurements were taken from distinct samples or whether the same sample was measured repeatedly
☐	☒	The statistical test(s) used AND whether they are one- or two-sided<br>*Only common tests should be described solely by name; describe more complex techniques in the Methods section.*
☐	☒	A description of all covariates tested
☐	☒	A description of any assumptions or corrections, such as tests of normality and adjustment for multiple comparisons
☐	☒	A full description of the statistical parameters including central tendency (e.g. means) or other basic estimates (e.g. regression coefficient) AND variation (e.g. standard deviation) or associated estimates of uncertainty (e.g. confidence intervals)
☐	☒	For null hypothesis testing, the test statistic (e.g. F, t, r) with confidence intervals, effect sizes, degrees of freedom and P value noted<br>*Give P values as exact values whenever suitable.*
☒	☐	For Bayesian analysis, information on the choice of priors and Markov chain Monte Carlo settings
☒	☐	For hierarchical and complex designs, identification of the appropriate level for tests and full reporting of outcomes
☒	☐	Estimates of effect sizes (e.g. Cohen's d, Pearson's r), indicating how they were calculated

Our web collection on statistics for biologists contains articles on many of the points above.

Software and code

Policy information about availability of computer code

Data collection	MaxQuant software (Version 1.0.13.13)<br>SpectroDive program (Version 8.0, Biognosys)<br>VisiView (Version 3.1.0.9)
Data analysis	FlowJo (V10)<br>GraphPad Prism V7<br>Fiji (ImageJ 1.51o)

For manuscripts utilizing custom algorithms or software that are central to the research but not yet described in published literature, software must be made available to editors/reviewers. We strongly encourage code deposition in a community repository (e.g. GitHub). See the Nature Research guidelines for submitting code & software for further information.

Data

Policy information about availability of data

All manuscripts must include a data availability statement. This statement should provide the following information, where applicable:

- Accession codes, unique identifiers, or web links for publicly available datasets
- A list of figures that have associated raw data
- A description of any restrictions on data availability

The authors declare that all data supporting the findings of this study are available from the corresponding authors upon request; the raw data will be uploaded to public database with accession codes.

Field-specific reporting

Please select the one below that is the best fit for your research. If you are not sure, read the appropriate sections before making your selection.

☒ Life sciences ☐ Behavioural & social sciences ☐ Ecological, evolutionary & environmental sciences

For a reference copy of the document with all sections, see nature.com/documents/nr-reporting-summary-flat.pdf

Life sciences study design

All studies must disclose on these points even when the disclosure is negative.

Sample size	Three independent biological replicates were performed for absolute quantification of protein copy number, protein abundance during time course of growth, and protein abundance after protein synthesis inhibition experiments. Three independent measurements were performed to determine the isotope-labeled peptide concentration in SpikeMix. Three independent biological replicates were performed for the cell enumeration.
Data exclusions	The search criteria for SRM LC-MS analysis were set as follows: full tryptic specificity was required (cleavage after lysine or arginine residues unless followed by proline), 3 missed cleavages were allowed, carbamidomethylation (C) was set as fixed modification and arginine (+10 Da), lysine (+8 Da) and oxidation (M) were set as a variable modification. The resulting msms.txt file was converted to a spectral library panel with the 5 to 10 best transitions for each peptide. Only elution groups with q-values < 0.01 were considered correct calls and used for quantification.
Replication	Measurements of peptide concentrations were performed for three times. Three independent biological replicates were performed for each experiment.
Randomization	no randomization
Blinding	investigators were not blinded.

Reporting for specific materials, systems and methods

We require information from authors about some types of materials, experimental systems and methods used in many studies. Here, indicate whether each material, system or method listed is relevant to your study. If you are not sure if a list item applies to your research, read the appropriate section before selecting a response.

Materials & experimental systems

n/a	Involved in the study
☒	☐ Antibodies
☒	☐ Eukaryotic cell lines
☒	☐ Palaeontology
☒	☐ Animals and other organisms
☒	☐ Human research participants
☒	☐ Clinical data

Methods

n/a	Involved in the study
☒	☐ ChIP-seq
☐	☒ Flow cytometry
☒	☐ MRI-based neuroimaging

Flow Cytometry

Plots

Confirm that:

☒ The axis labels state the marker and fluorochrome used (e.g. CD4-FITC).

☒ The axis scales are clearly visible. Include numbers along axes only for bottom left plot of group (a 'group' is an analysis of identical markers).

☒ All plots are contour plots with outliers or pseudocolor plots.

☒ A numerical value for number of cells or percentage (with statistics) is provided.

Methodology

Sample preparation	bacterial cells (Pseudomonas aeruginosa PAO1, Vibrio cholerae 2740-80 and Acinetobacter baylyi ADP1) were used.
Instrument	FACS-Canto-II
Software	FlowJo V10

Cell population abundance	in total 50000 events were recorded for each sample.
Gating strategy	Cells and beads were gated on FSC-H vs Ex488_LP502_BP530_30_H (green fluorescence). P. aeruginosa cells are counts with FSC-H between 50-1000 and Ex488_LP502_BP530_30_H above 100; V. cholerae cells are counts with FSC-H between 50-1000 and Ex488_LP502_BP530_30_H above 500; A. baylyi cells re counts with FSC-H between 100-10000 and Ex488_LP502_BP530_30_H above 500. Beads are counts with FSC-H above 20000 and Ex488_LP502_BP530_30_H above 100.

☒ Tick this box to confirm that a figure exemplifying the gating strategy is provided in the Supplementary Information.
